# The Magnitude and Associated Factors of Early Index Case Testing Among Adult HIV Index Cases at Debre Markos Town High Load Health Facilities 2023

**DOI:** 10.1155/arat/8237131

**Published:** 2025-06-27

**Authors:** Dessie Tarekegn, Samuel Derbie Habtegiorgis, Animut Takele Telayneh, Kalkidan Worku Mitiku, Adane Adugna, Dawit Alemayehu, Muluken Teshome

**Affiliations:** ^1^Department of Internal Medicine, Debre Markos Comprehensive Specialized Hospital, Debre Markos, Ethiopia; ^2^Department of Public Health, Debre Markos University, Debre Markos, Ethiopia; ^3^Department of Medical Laboratory Science, Debre Markos University, Debre Markos, Ethiopia; ^4^Department of Surgery, Debre Markos University, Debre Markos, Ethiopia

**Keywords:** adult, Debre Markos, early index case testing, Ethiopia, index case

## Abstract

**Introduction:** Early human immunodeficiency virus (HIV) testing of partners or families of the index case is an innovative type of testing which is performed within 14 days after contact elicitation using a contractual referral approach. Testing within this time is very important because it helps to identify contacts early and enroll them for intervention timely. In most health facilities, the contractual approach was not practiced; by implementing this early index case testing, most contacts could be tested early and it could prevent them from HIV-related death.

**Objective:** This study aimed to assess the magnitude and associated factors of early index case testing among adult HIV index cases in Debre Markos town high case load health facilities.

**Methods:** Institution-based cross-sectional study was conducted on adult index cases who started antiretroviral therapy from December 1/2018 to August 30/2022. A total of 384 index case charts were selected by the systematic random sampling technique using their medical record number from January 23/2023 up to February 28/2023. After the data were collected using a structured checklist, they were entered into EpiData Version 4.6 and then exported to SPSS software Version 25 for data cleaning, coding, categorizing, and further analysis. The Hosmer–Lemeshow test goodness of fit was checked for model fitness. Both bivariable and multivariable logistic regression analyses were used to identify significant factors with early testing of index cases' families. Finally, variables having *p*value < 0.05 with 95% CI in the multivariable logistic regression are considered as significant factors.

**Result:** In this study, the magnitude of early index case testing among adult index patients was 28.6% (95% CI: 27.17%–30.0%). Being female (AOR = 1.89, 95% CI (1.17–3.06)), urban resident index cases (AOR = 1.88, 95% CI (1.16–3.03)), and having disclosure status (AOR = 2.34, 95% CI (1.40–3.92)) were significantly associated with early index case testing.

**Conclusion:** This study examines the prevalence and key factors influencing early index case HIV testing among adult patients in Debre Markos town. By identifying critical determinants such as gender, residence, and disclosure status, it provides valuable insights into how early testing can be enhanced to reduce transmission and improve health outcomes.

## 1. Introduction

The most advanced stage of human immunodeficiency virus (HIV) infection, known as acquired immunodeficiency syndrome (AIDS), is characterized by the emergence of opportunistic infections (OIs), various severe clinical symptoms, and certain malignancies. When index cases are enrolled in chronic care and treatment, early index case testing is one sort of HIV testing approach that helps them elicit their untested partner and family members, and it is the most essential manner of tracing strategy to locate infected index contacts [1]. The global scale-up of early index case testing represents a significant achievement, as it increases access to testing for family members of index cases and contributes to the reduction of new infections, morbidity, and mortality. But the progress is low, which is only 30% in 2016 [2]. The World Health Organization (WHO) recommended several HIV testing approaches used in different countries, including provider-initiated testing and counseling (PITC), voluntary counseling and testing (VCT), partner and family-based index case testing (P&FB-ICT), HIV self-testing (HIVST), and social network–based HIV testing (SNS) [1]. In Ethiopia, under index case testing, there are four HIV testing approaches for partners and family members; these are client referral approach, contractual referral approach, dual referral approach, and provider referral approach [3].

In different countries, the P&FB-ICT is used as mainstay prevention of HIV [4, 5]; this is because of the high HIV transmission and positivity rate among partners and family members of index case [6]. A new set of targets was released by UNAIDS in December 2020 which states that by 2025, 95% of all individuals living with HIV will be aware of their status, 95% of those diagnosed with the virus will receive continuous antiretroviral therapy (ART), and 95% of those on therapy will have viral suppression. In 2021, all United Nations member states adopted strategies to combat the impact of HIV/AIDS [7]. Globally, there are 37.9 million people living with HIV (PLHIV); only 84% of individuals had known their HIV status in 2020. In the same year, around 1.5 million people were newly infected with HIV, and 680,000 people died from HIV/AIDS-related illnesses [3]. By 2020, the prevalence of HIV/AIDS varied throughout Ethiopia; in Somalia, it is 0.15%, while in Gambela, it is 4.13% [8]. To meet the target objectives, nationally further effort is required to improve HIV case identification performance and achieve epidemic control [7].

Early index case testing is influenced by a variety of index case testing factors. Female index cases [9], children of HIV positive family [10–12], elicited sexual/biological contacts [13, 14], knowledge of the testing approach (index case testing) [14, 15]. The impact of delayed of early index case testing for index case contacts after elicited can affect their health condition and the relative benefit of earlier testing [16]. Early elicitation, diagnosis and early treatment can be reducing high morbidity and mortality from HIV related OIs [17].

Research indicates that the incidence of HIV/AIDS among partners and family members of index cases is significantly high, approaching 33% [18–20]. Although the incidence of HIV/AIDS is more prevalent in indexed case family, P&FB-ICT is not satisfactory, especially in African countries, including Ethiopia [2, 5, 21–26]. Using innovative contractual testing approaches for partners and family members of index cases facilitates early access to prevention, care, and treatment. However, implementing such innovative approaches may present significant challenges [27].

Early index case testing using contractual referral approach for family members is low and it is a major health burden. Most studies conducted in Ethiopia were not focused on early index case testing for index cases, sexual or biological contacts.

Thus, this study aimed to fill the knowledge gap about the extent of early index case testing and to identify factors associated with adult index case testing.

## 2. Methods

### 2.1. Study Design, Area, and Period

An institutional-based cross-sectional study design was conducted. The study was conducted at Debre Markos Comprehensive Specialized Hospital and Debre Markos Health Center, in areas with a high case load of ART health facilities. Debre Markos town is located 300 km from Addis Ababa, the capital city of Ethiopia, and 265 km from Bahir Dar, the capital city of Amhara National Regional State. There are 8703 clients ever enrolled on ART since the service started in both health facilities. Currently, including transfer out (TO) and interruption in treatment (IIT), there are 3,685 index cases at Debre Markos Comprehensive Specialized Hospital and 1,300 index cases at Debre Markos Health Center receiving follow-up care in the ART and PMTCT clinics. Among these patients, 4,853 are adults and 132 are pediatric patients under 15 years old who are taking ART [28]. The study includes the data of clients who have been enrolled to chronic HIV care and treatment within a period from December 1/2018 to August 30/2022, and the data were extracted from January to February 2023.

### 2.2. Source Population

Source population included adult index cases on chronic HIV care at Debre Markos town government health facilities enrolled between December 1/2018 and Aug 30/2022.

### 2.3. Study Population

Study population included all adult index cases who were enrolled to ART care and treatment during the study period from December 1/2018 to August 30/2022 at Debre Markos Comprehensive Specialized Hospital and Debre Markos Health Center.

### 2.4. Eligibility Criteria

#### 2.4.1. Inclusion Criteria

Inclusion criteria included all adult index cases who had elicited partners and family members on the ICT register since December 1/2018 to August 30/2022.

#### 2.4.2. Exclusion Criteria

Adult index cases who had incomplete data or variables (like no date of contact elicited, no date of contact HIV tested, and index chart) were excluded.

### 2.5. Sample Size Determination and Sampling Technique

#### 2.5.1. Sample Size Determination

The required sample size was calculated based on two specific objectives. For the first objective, the single population proportion formula was used, considering the following assumptions:

(1) $$\begin{array}{c} n=\frac{\left.{\left(Za/2\right)}^{2}\right)P\left(1-P\right)}{d^{2}}. \end{array}$$

*n* = minimum sample size required for the study.

*Z* = standard normal distribution with confidence interval of 95%, *Za*/2 = 1.96.

*P* = the proportion of HIV testing among elicited family members.

*d* = marginal error.

The sample size for the first objective was calculated by taking *p* as 49.3% [29].

(2) $$\begin{array}{c} n=\frac{{\left(1.96\right)}^{2}\times \left(0.493\right)\times \left(0.507\right)}{{\left(0.05\right)}^{2}},\;n=384. \end{array}$$

For the second objective, the sample size was determined by using Epi Info Stat Calc software by 95% CI with 5% margin of error, 80% power, and 1 to 1 ratio of exposed to unexposed. But from the calculated sample size, the first objective is large and taken as a fine sample size for the study as 384.

#### 2.5.2. Sampling Technique

**Figure 1:**
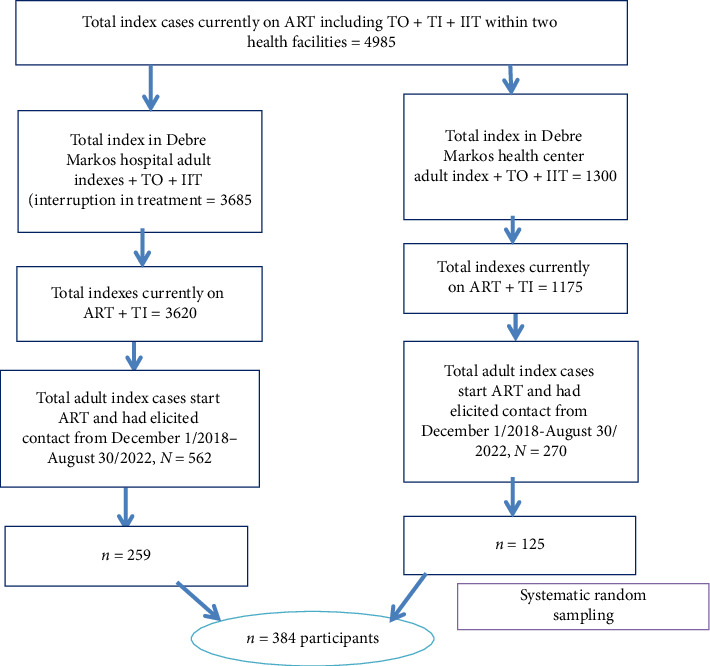
Schematic presentation of sampling procedure of early index case testing and associated factors among adult index cases attending ART clinic at Debre Markos Comprehensive Specialized Hospital and Debre Markos Health Center in East Gojjam Zone, Northwest Ethiopia, 2023.

Systematic sample was implemented after subjects proportionally allocated for two institutions. The sampling fraction *k* is computed for two institutions separately, as follows. For Debre Markos hospitals, *k* = 562/259 ≈ 2; for Debre Markos health center, *k* = 270/125 ≈ 2. The first participant was chosen by considering lottery method of *k* (1 − 2), and the subsequent participants were chosen at intervals of every *k* value. Sampling was implemented for two institutions separately (Figure 1).

### 2.6. Variables

#### 2.6.1. Dependent Variables

Early index case testing (Yes, No).

#### 2.6.2. Independent Variables

Sociodemographic factors: Age, sex, religion, marital status, occupation, residence, educational status, transportation access, and member of PLHIV association.

ART and clinical information of index: Functional status, duration on ART, adherence level, WHO staging, disclosure status, and offering ICT service. Individual factors: Having a cell phone, awareness about ICT service, and living with family members.

#### 2.6.3. Operational Definition

Index case: A client who is newly diagnosed as HIV-positive and enrolled in HIV care and treatment services [25].

Adult index cases: Index cases who are on chronic HIV care aged 15 years and above [2].

Early index case testing: A voluntary process in which ART healthcare providers ask index cases to elicit all their partners, biological children, siblings, and other relatives and conduct HIV testing using a contractual referral approach within 14 days [2].

Contractual referral approach: The index client enters into a “contract” with the counselor whereby they agree to bring their partners or family members to HIV testing service (HTS) within 14 days [30].

Spouse partner: Partners not separated or divorced living in the same household with index cases [25].

Family members: People living in the household, consisting of sexual partners, biological children, and other members connected with them either biologically or by choice.

High caseload health facilities: Those ART service delivery health facilities have more than 500 PLHIV in ART and PMTCT clinics [3].

Elicitation: Process of listing down each family member and partner of index cases after offering the index cases about ICT at the time of ART initiation.

Offering ICT service: Provide comprehensive information to index cases about partner and family-based index case testing, ensuring they understand their rights [2].

### 2.7. Data Collection Procedures and Tools

A data collection checklist was developed to capture secondary data from the index case testing register, the electronic medical record (EMR) smart care system, and client charts in the ART and PMTCT clinics. The checklist format was adapted from the previous related literature and modified to fit the index case charts and the facility's EMR-based smart care system, ensuring all necessary data could be collected. For this purpose, two BSc degree holders with nursing experience in the area extracted the required data from charts and registers. Additionally, two card room workers were responsible for retrieving the charts, and two data clerks managed data extraction from the electronic smart care system. These personnel were assigned to two health facilities for the entire data collection period. In addition, 2 BSc holders with nursing for two health facilities who were supposed to manage the overall data collection were deployed. The overall data collection was taken in a month and was monitored and cascaded by the principal investigator.

A consent form was filled up and signed by the heads of the organization before any data gathering began. The leaders of the institutions signed a declaration indicating their consent to utilize patient data for research purposes after reading and comprehending the study's purpose.

### 2.8. Data Quality Control

A one-day training was provided to two data collectors, two data clerks, two card room workers, and two supervisors. The training covered procedures for retrieving client charts and registers, extracting data, identifying source documents, and understanding the scope of the study. The data extraction form was reviewed and commented on by ART care provider experts in the field to ensure its consistency and relevance. Strict follow-up and supervision were carried out during data collection. The principal investigator and supervisors conducted day-to-day follow-up during the whole period of data collection. Each data extraction form was reviewed and checked for completeness by the principal investigator and supervisor. The necessary feedback was given to data collectors and others who were involved. The principal investigator was supervising the overall activity of the data collection.

### 2.9. Data Management and Analysis

The extracted data were coded and entered into EpiData Version 4.6, then checked for completeness, and cleaned to check for frequencies, accuracy, consistencies, and missed values and variables. The data were exported from EpiData to SPSS Version 25 for analysis. Then the data were categorized and sorted to facilitate their analysis.

The percentage and frequency of the patients about all variables were summarized by descriptive statistics, and cross-tabulations were performed for the descriptive data. Tables, figures, and texts were used for data presentation. Variables in the binary logistic regression analysis with *p* values ≤ 0.25 were entered into the multivariable analysis. Multicollinearity was checked by a variance inflation factor (VIF). Model fitness was checked by using the Hosmer–Lemeshow goodness of fit (the *p*value was 0.40). Finally, variables having a *p* value less than 0.05 at a 95% confidence interval in multivariable analysis were considered as significant factors.

## 3. Results

### 3.1. Sociodemographic Characteristics of Participants

Three hundred eighty-four participants' charts were reviewed for this study. Among the study participants, 166 (43.2%) were males. The majority age group (41.4%) was 35–45 years, and concerning the marital status of the clients, nearly half of 186 (48.4%) were single. Based on their religion distribution, more than four-fifths (85.9%) were Orthodox. One-third of clients (35.4%) were from urban residences (Table 1).

### 3.2. Index Case Clinical and ART Characteristics

The majority of participants (95.3%) had a duration on ART of more than 12 months, and 75% were WHO clinical stages 1 and 2. Regarding their functional status, nearly four-fifths (79.2%) were working. More than one-fourth (27.9%) had interruption of ART. Almost two-fifths (39.1%) of participants disclosed their HIV results to their sexual and biological contacts. More than three-fifths (65.4%) of participants had good adherence to ART, and 72.9% of participants were offered ICT service (Table 2).

### 3.3. Individual-Related Characteristics of Participants

More than half (55.7%) of the participants had cell phones, and 51.8% of participants were living with their partners or family members. Almost half (49.0%) of the participants had index case testing service awareness.

### 3.4. Magnitude of Early Index Case Testing

The magnitude of early index case testing among adult index cases was 28.6% (95% CI: 27.17%−30.0%).

### 3.5. Factors Associated With Early Index Case Testing

In bivariable logistic analysis, variables with *p* value ≤ 0.25 were candidates for multivariable logistic regression analysis. Sex, residence, WHO clinical stage, member of PLHIV association, interruption of ART drug, disclosure status, ART adherence level, offering ICT service, and being an owner of a cell phone were candidates for multivariable analysis.

In the final model, being female, having an urban residence, and having disclosure status were identified as significantly associated factors for early index case testing in multivariable logistic regression analysis.

Female index cases were 89% more likely than male index cases to bring their partners or family members for early index case testing (AOR = 1.89, 95% CI [1.17–3.06]). Those index cases who live in urban were 88% (AOR = 1.88, 95% CI [1.16–3.03]) more likely to bring their family members for early index case testing as compared to rural residents. Additionally, index cases who had disclosed their HIV status were 2.34 times more likely to bring their family members for early index case testing compared to those who had not disclosed their status (AOR = 2.34, 95% CI [1.40–3.92]) (Table 3).

## 4. Discussion

In this study, the magnitude of early index case testing was 28.6% (95% CI: 27.17%−30.0%). The finding aligns with a related study conducted in Eastern China (30%) [31], Ivory Coast (30%) [32], and Rwanda [33]. However, the finding of this study was lower than that of the study done in the United Kingdom (43.6%) [34]. The possible explanation for the above variation could be due to the study population characteristics and the difference in the tool used to measure early HIV testing. Other possible explanation might be difference in health service accessibility, community awareness about early testing, the culture of stigma and disclosing, and the healthcare worker availability skills and engagement difference. The HIV testing and medication accessibility may also be another variation. This study finding was also lower than that of the studies conducted in Lusaka, Zambia (52%) [21], Kenya (97%) [35], and Addis Ababa, Ethiopia (38%) [13]; this variation may be due to the difference in study areas and socioeconomic status of index cases. In other ways, this finding was higher than that of a study done in San Francisco (27%) [36] and a study conducted in South Africa (10.3%) [9]. This variation may be due to the difference in study areas, the study period, the socioeconomic status of participants, times of study done, and variations in the tools used to assess early index case testing.

The odds of female index cases were 1.89 times more likely had brought their partners or family members for early index case testing than male index cases (AOR = 1.89, 95% CI (1.17–3.06)). This finding was in agreement with studies conducted in South Africa [37], Malawi [38] and Addis Ababa University College of Health Science Ethiopia [25]. The possible reasons for this result might be females are early accepters, fear of illness, fear of their children being ill and they can cope with stigma and discrimination in the community.

Index case participants from urban areas were 88% more likely to bring their partners or family members for early index case testing compared to those from rural areas (AOR = 1.88, 95% CI 1.16–3.03). This finding was supported by a related study done in Eastern Africa [39] and in Kenya [40]. The possible reasons for this result could be transportation access, level of education of the participant, work condition of the participant, near to information, utilization of mass media, and geographical distance from health facility.

This study found that participants who had disclosed their HIV status were 2.34 times more likely to bring their partners or family members for early index case testing compared to those who had not disclosed their status (AOR = 2.34, 95% CI 1.40–3.92). This finding was supported by a study conducted in Malawi [17] and Ethiopia [29, 41, 42]. This finding may be attributed to the fact that disclosure is highly important for index cases receiving HIV care, as it provides several benefits for their family members. These include reducing psychological stress, enabling exposed partners and children to undergo HIV testing, and lowering the risk of future HIV transmission through early diagnosis. In addition, disclosure facilitates good behaviors that can improve the management and treatment of HIV; women who disclose their status to their partners could be more likely to participate in the PMTCT service and get healthy children [43].

Many index cases failed to inform their partners and family members of their HIV status at an early stage, despite receiving extensive counseling regarding the significance of disclosing HIV results to them during partner and family elicitation before HIV testing. Thus, early index case testing process communication of HIV test results to their partners and family members is very important [44].

Another study demonstrates that throughout the elicitation process of biological or sexual connections of index cases, the main worry is effective disclosure awareness of index cases about P&FB-ICT services because it might enhance and encourage early index case testing for their intimate or biological relationships [41].

## 5. Conclusion

Out of all the index cases in this study, 28.6% took their partners and family members for early index case testing. Disclosure status, residence, and gender were significant factors for early index case testing.

We recommend strengthening early index case testing through strong mutual understanding counseling and a friendly approach. It is also better to expand the awareness and education through local media, community engagement, and cultural ceremonies and encourage the local key and respected people like religious leaders, community leaders, and influencers to lead the awareness creation. Researchers may conduct future research with a large sample size and include more variables. Additionally, they may include qualitative methods to identify barriers and motivations influencing early testing from patient perspectives. This research provides important insights into the challenges and opportunities for implementing index case testing more effectively, particularly when considering the health system's resources, accessibility, and cultural barriers and facilitators.

## Figures and Tables

**Table 1:** Sociodemographic characteristics of adult index cases in Debre Markos Comprehensive Specialized Hospital and Debre Markos Health Center, Northwest Ethiopia, 2023 (*N* = 384).

Sociodemographic	Categories	Frequency	Percent (%)
Sex	Male	166	43.2
	Female	218	56.8

Age	15–24 years	14	3.6
	25–34 years	105	27.3
	35–45 years	159	41.4
	> 45 years	106	27.6

Marital status	Single	186	48.4
	Married	68	17.7
	Divorce and widowed	130	33.9

Residence	Urban	136	35.4
	Rural	248	64.6

Educational status	College and above	141	36.7
	Secondary education (G9-12)	100	26.0
	Primary education	80	20.3
	Unable to read and write	67	16.9

Religion	Orthodox	330	85.9
	Muslim	19	4.9
	Protestant	35	9.1

**Table 2:** Index cases' ART and clinical characteristics, Debre Markos Comprehensive Specialized Hospital and Debre Markos Health Center, Northwest Ethiopia, 2023 (*N* = 384).

Sociodemographic	Categories	Frequency	Percent (%)
Month on ART	< 12 months	18	4.7
	> 12 months	366	95.3

WHO stage	Stages 1 and 2	288	75
	Stages 3 and 4	96	25

Functional status	Working	304	79.2
	Ambulatory	65	16.9
	Bedridden	15	3.9

Interruption of ART	Yes	107	27.9
	No	277	72.1

Disclosure	Yes	150	39.1
	No	234	60.9

Adherence	Good	251	65.4
	Poor and fair	133	34.6

**Table 3:** Factors associated with early index case testing among adult index cases in Debre Markos public health institutions, Northwest Ethiopia, 2023 (*n* = 384).

Variables	Categories	Early index case testing		COR (95% CI)	AOR (95% CI)	*p* value
		Yes	No			
Sex	Female	73	145	1.75 [1.1–2.8]	**1.89**[1.2 − 3.1]^∗^	0.01
	Male	37	129	1	1	

Residence	Urban	50	86	1.82 [1.2–2.9]	**1.88**[1.2 − 3.0]^∗^	0.01
	Rural	60	188	1	1	

WHO stage	1 and 2	88	200	1.48 [0.9–2.5]	0.59 [0.3–1.1]	0.08
	3 and 4	22	74	1	1	

Member of association	Yes	59	171	0.69 [0.4–1.1]	1.27 [0.8–2.0]	0.32
	No	51	103	1	1	

Interruption of ART	Yes	25	82	1	1	
	No	85	192	0.68 [0.4–1.2]	0.62 [0.4–1.1]	0.10

Disclosure status	Yes	53	97	1.69 [1.1–2.7]	**2.34**[1.4 − 3.9]^∗^	0.01
	No	57	177	1	1	

Adherence status	Good	77	174	1.34 [0.8–2.2]	0.73 [0.4–1.3]	0.26
	Fair and poor	33	100	1	1	

Offered ICT	Yes	85	195	1.37 [0.8–2.3]	0.74 [0.4–1.3]	0.31
	No	25	79	1	1	

Has index case cell phone	Yes	67	147	1.34 [0.9–2.1]	0.76 [0.5–1.2]	0.28
	No	43	127	1	1	

*Note:* Bold values represent *p* value < 0.01.

^∗^Significant at AOR with *p* value< 0.05 and 1 = reference.

## Data Availability

The data that support the findings of this study are available on request from the corresponding author. The data are not publicly available due to privacy or ethical restrictions.

## Nomenclature

ART: Antiretroviral therapy
DMCSH: Debre Markos Comprehensive Specialized Hospital
DMHC: Debre Markos Health Center
EMR: Electronic medical record
HCPs: Healthcare providers
HTS: HIV testing service
IC: Index case
ICT: Index case testing
IIT: Interruption in treatment
PITC: Provider-initiated testing and counseling
PLHIV: People living with HIV
PMTCT: Prevention of mother to child transmission
SNS: Social network service
TO: Transfer out
VCT: Voluntary counseling and testing
WHO: World Health Organization
